# Trigeminal nerve-driven neurogenic inflammation linking migraine to glioblastoma invasion: a literature review

**DOI:** 10.3389/fimmu.2025.1632154

**Published:** 2025-07-16

**Authors:** Xiaoli Song, Qian Zhu, Jieying Zhang, Jin Yang, Xinxin Zhang, Qian Song

**Affiliations:** ^1^ National Clinical Research Center for Chinese Medicine Acupuncture and Moxibustion, Tianjin, China; ^2^ First Teaching Hospital of Tianjin University of Traditional Chinese Medicine, Tianjin, China

**Keywords:** migraine, glioblastoma, trigeminal nerve, neurogenic inflammation, neuropeptides

## Abstract

Migraines are among the most common neurological disorders, disabling nearly one in seven people worldwide, whereas glioblastoma (GBM) is the most aggressive primary brain tumour, with median survival scarcely beyond 15 months. Historically considered distinct, these conditions are increasingly linked by trigeminal nerve-driven neurogenic inflammation. Activation of trigeminovascular afferents provokes antidromic release of calcitonin gene-related peptide (CGRP), substance P (SP), and pituitary adenylate cyclase-activating polypeptide (PACAP); beyond mediating migraine pain, these peptides remodel vasculature, immune infiltrates, and extracellular matrix to facilitate GBM invasion. Pre-clinical studies show CGRP and SP up-regulate matrix-metalloproteinases and integrins, while PACAP modulates cAMP–MAPK signalling, collectively promoting perivascular migration and temozolomide resistance. Epidemiological analyses report higher migraine antecedents in patients later diagnosed with brain tumours, and high-resolution MRI frequently localises GBM spread along trigeminal pathways, underscoring anatomical plausibility. Emerging therapeutics mirror these insights: aprepitant (an NK1-receptor antagonist) triggers GBM apoptosis, gepant-class CGRP blockers curb invasive phenotypes, and radiolabelled SP analogues deliver focal alpha-therapy. These discoveries facilitate more precise pathogenetic characterisation, reduce diagnostic uncertainty, and expedite translational drug development. This review synthesises current evidence on trigeminal neurogenic inflammation as a mechanistic conduit between migraine biology and GBM progression, mapping cellular circuits, molecular crosstalk, and translational interventions. By integrating neurobiology, oncology, and pharmacology, we aim to delineate diagnostic blind spots, spotlight drug-repurposing opportunities, and chart a roadmap toward personalised strategies that simultaneously alleviate migraine burden and restrain glioblastoma aggressiveness.

## 1 Introduction

Both migraine and glioma contribute substantially to global neurological disability (1, 2). Migraine alone afflicts roughly one in seven individuals and stands among the leading contributors to neurological years-lived-with-disability globally (3–5). Glioblastoma (GBM), the most common lethal brain tumour, still carries a median survival of only ~15 months despite multimodal therapy (6, 7). Ipsilateral tumour-headache and incidental benefit of anti-migraine drugs in GBM therapy highlight neuron–tumour crosstalk unique to cranial, not peripheral, cancers (8–10).

Central to migraine pathogenesis is trigeminovascular neurogenic inflammation. Electrical or chemical activation of trigeminal nociceptors provokes a rapid antidromic release of vasoactive neuropeptides—chiefly calcitonin gene-related peptide (CGRP), substance P (SP), and pituitary adenylate cyclase-activating peptide (PACAP)—from perivascular afferents innervating the dura and cortical vessels (11, 12). CGRP’s importance is underscored by the dense expression of its canonical receptor components, calcitonin receptor-like receptor (CLR) and receptor activity-modifying protein-1 (RAMP1), on human trigeminal ganglion neurons, satellite glia, and vascular smooth-muscle cells (13–15). These peptides collectively induce vasodilatation, plasma protein extravasation, mast-cell degranulation, and leukocyte chemotaxis—hallmarks of the sterile inflammatory milieu that sensitises meningeal nociceptors and drives migraine pain (16–18).

Intriguingly, each of these neuropeptides also possesses documented oncological bio-activity within the glioma micro-environment (19–21). PACAP and vasoactive intestinal peptide (VIP) modulate cyclic-AMP signalling and proliferation in C6 and T98G glioma models, revealing cell-context–dependent pro- and anti-growth actions. Substance P engages the neurokinin-1 receptor (NK1-R), which is consistently over-expressed across the glioma malignancy spectrum and has become a target for radionuclide therapy in recurrent GBM (22–24). Beyond direct mitogenic effects, neuropeptide stimulation enhances extracellular-matrix remodelling: CGRP and SP up-regulate matrix-metalloproteinases (MMP-2, MMP-9) and membrane-type MMPs that are indispensable for GBM cell invasion along white-matter tracts (25–27). These observations point to a molecular convergence whereby trigeminal-derived mediators, originally evolved for host defence and vasoregulation, inadvertently cultivate a micro-environment permissive to glioma infiltration.

Recognising this overlap, the present review interrogates the hypothesis that trigeminal nerve-driven neurogenic inflammation constitutes a mechanistic bridge between migraine biology and GBM progression. We first dissect the cellular and molecular architecture of trigeminal neurogenic inflammation in migraine, then map how the same mediators and signalling nodes orchestrate GBM invasion. By integrating otherwise disparate literatures, we aim to illuminate novel pathophysiological cross-talk and identify therapeutic targets capable of attenuating both migraine burden and glioblastoma aggressiveness.

## 2 Trigeminal neurogenic inflammation in migraine

**Figure 1:**
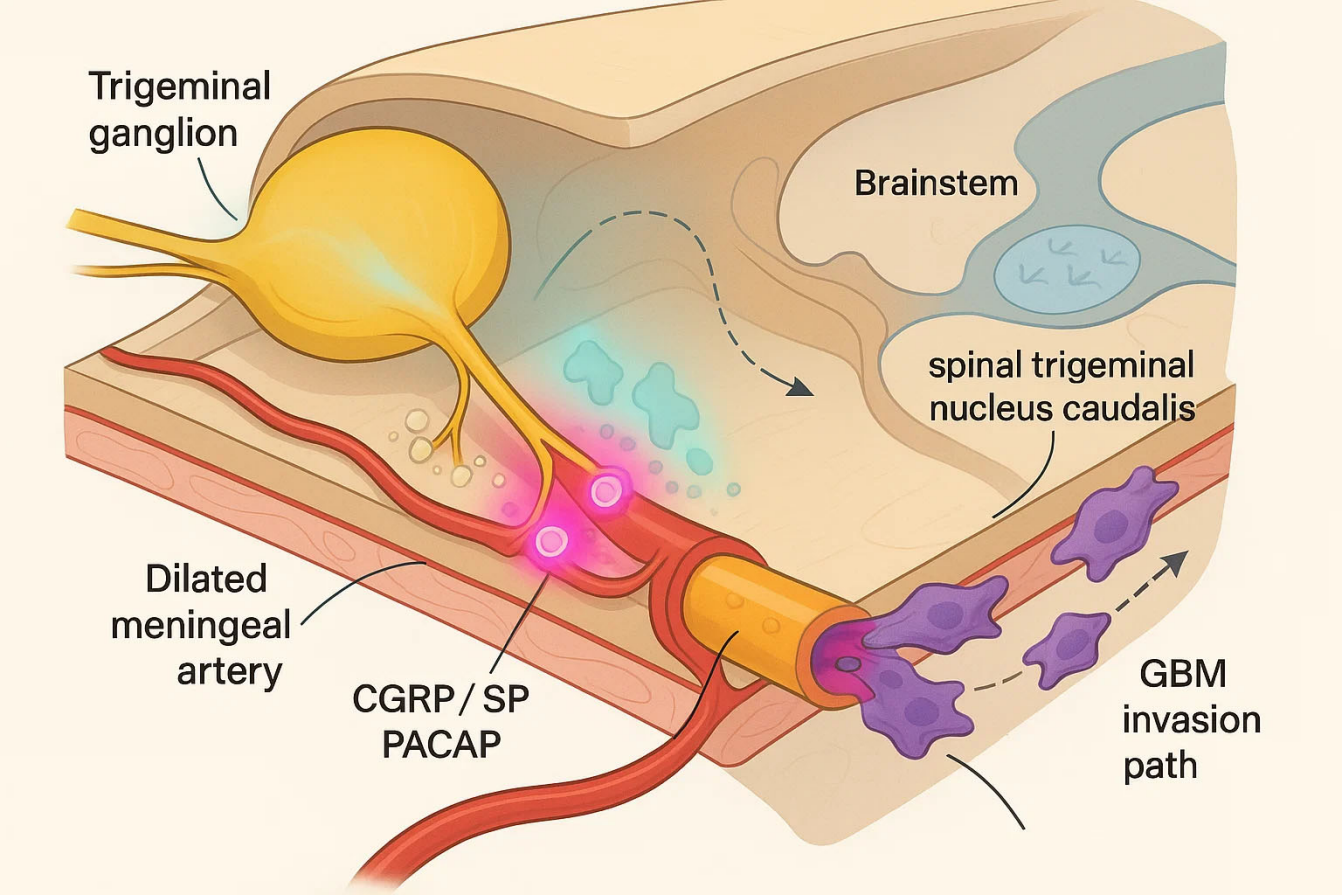
Trigeminovascular neurogenic inflammation as a bridge between migraine and glioblastoma invasion.

As shown in **Figure 1**, the trigeminovascular system (TVS) forms an anatomically discrete, yet functionally expansive, pain-signalling network in which pseudo-unipolar neurons of the ophthalmic branch of the trigeminal nerve project collaterals to both cranial vessels and the spinal trigeminal nucleus caudalis (28, 29). These perivascular afferents densely invest the dura mater, middle-meningeal artery and cortical arterioles, positioning the TVS to couple vascular status with nociceptive traffic. Electrical, mechanical or metabolite-driven activation of meningeal C- and Aδ-fibres—whether secondary to cortical spreading depolarisation, meningeal stretch or nitrosative stress—initiates a robust antidromic secretory reflex that is now recognised as the biochemical fulcrum of migraine pain (30–32).

Within milliseconds of depolarisation, large-dense-core vesicles at the peripheral terminals fuse and discharge a stereotyped cocktail of neuropeptides (33). Approximately two-thirds of human trigeminal ganglion neurons co-express calcitonin gene-related peptide (CGRP) and substance P (SP), while 15–20% harbour pituitary adenylate cyclase-activating polypeptide-38 (PACAP-38) or its splice variants (34, 35); immunofluorescence and *in-situ* hybridisation in rodent and human tissues confirm parallel expression of their canonical receptors (CLR/RAMP1 for CGRP, NK1-R for SP, PAC1-R/VIP-R1 for PACAP/VIP) on satellite glia, vascular smooth-muscle cells and meningeal fibroblasts (36, 37). PACAP-38 itself can provoke secondary CGRP release from both ganglion and central terminals, illustrating hierarchical neuropeptide crosstalk that amplifies the inflammatory signal. CGRP, together with nitric-oxide–cGMP signalling, is the principal effector in this cascade. Nanomolar concentrations dilate cranial arteries via cyclic-AMP–dependent protein kinase and endothelial nitric-oxide synthase, while concurrently lowering nociceptor firing thresholds through CaV3.2 T-type channel modulation (38, 39). Genomic data show higher RAMP1/CALCA expression in migraineurs; oestrogen-responsive enhancers and X-chromosome copy-number gains, together with Y-linked TSPY loss, amplify CGRP output from hormone-primed trigeminal and GBM cells (40, 41). SP complements these actions by engaging endothelial NK1-receptors to increase post-capillary venular permeability and provoke plasma-protein extravasation, a hallmark of sterile neurogenic inflammation visualised in rodent dura and attenuated by triptans or NK1 antagonists.

The inflammatory milieu that ensues is not neuron-restricted. SP and CGRP trigger rapid degranulation of dural mast cells; histamine, tumour-necrosis factor-α and tryptase released thereby further sensitise afferents and recruit neutrophils and macrophages, perpetuating the cycle (42, 43). Parallel activation of meningeal fibroblasts and endothelial cells leads to interleukin-6 and prostanoid synthesis, creating a cytokine gradient that diffuses centrally and primes second-order neurons (44, 45).

Central amplification follows. Persistent primary-afferent barrage phosphorylates NMDA receptors and ERK1/2 within the spinal trigeminal nucleus caudalis; activated microglia and astrocytes liberate brain-derived neurotrophic factor and nitric oxide, sustaining long-term potentiation of nociceptive neurons and manifesting clinically as cutaneous allodynia (13, 46). Functional imaging corroborates these findings, and both attacks and CGRP/PACAP release peak in the early morning, hinting at circadian control (29, 47).

Trigeminal neurogenic inflammation represents a dynamic, multi-cellular feed-forward loop in which neuropeptide release, vascular dysfunction, immune cell mobilisation and central sensitisation operate in concert. This finely tuned yet pathologically labile circuitry not only underpins migraine pain but also generates a repertoire of cytokines, proteases and growth factors that reshape the local extracellular matrix. Many of these same mediators—CGRP-driven matrix metalloproteinase induction, SP/NK1-R–mediated mitogenic signalling and PACAP-dependent cyclic-AMP modulation—are co-opted by glioblastoma cells to infiltrate neural parenchyma. Understanding the bidirectional dialogue between trigeminal afferents and their vascular–immune partners therefore offers a conceptual bridge between episodic migraine and malignant glioma invasion.

## 3 Molecular convergence driving glioblastoma invasion

The same neuropeptide circuits that ignite trigeminal neurogenic inflammation are increasingly recognised as oncogenic drivers within the glioblastoma (GBM) micro-environment. Transcriptomic and single-cell atlases show that high-grade gliomas over-express both tachykinin and calcitonin-family G-protein-coupled receptors (GPCRs); notably, neurokinin-1 receptor (NK1-R) and the calcitonin receptor-like receptor (CLR, encoded by CALCRL) track with mesenchymal programmes and shortened survival, underscoring their functional relevance to tumour spread (12, 48).

Substance P (SP) signalling exemplifies this hijacking. Matrix metalloproteinases (MMPs) are zinc-dependent endopeptidases that cleave basement-membrane type IV collagen, laminin and proteoglycans; the gelatinases MMP-2 and MMP-9 become fully active when surface-trimmed by the membrane-type protease MT1-MMP (MMP-14) and are opposed by tissue inhibitors of metalloproteinases (TIMP-1/-2). This triad is indispensable for GBM cells to tunnel along myelinated white-matter tracts. GBM cells form an autocrine loop in which neuronal or tumour-derived SP engages NK1-R to activate ERK1/2 and PI3K–Akt, culminating in β-arrestin-1 recruitment, cyclin‐dependent kinase activation and accelerated cell-cycle transit. Pharmacologic or genetic blockade of NK1-R curtails SP-driven chemotaxis, while the clinically approved antagonist aprepitant suppresses lamellipodia dynamics and reduces orthotopic tumour burden *in vivo* (49–51). Mechanistically, NK1-R stimulation up-regulates matrix-metalloproteinase-2 (MMP-2) and its membrane activator MT1-MMP, thereby enabling perivascular and white-matter tract infiltration (52).

A parallel axis operates through CGRP and its receptor family. Glioblastoma specimens and cell lines display heightened CLR/RAMP2–3 expression, a configuration better known as the adrenomedullin (ADM) receptor. ADM and CGRP ligation elevates intracellular cAMP, trans-activates JNK, and drives cyclin-D1–dependent proliferation; neutralising ADM antibodies or silencing CLR attenuate spheroid outgrowth and invasion in xenograft models (53–55). Moreover, CALCRL co-expresses with SERPINE1 and MMP-14 on invasive edge populations, linking CGRP-family signalling to extracellular-matrix turnover (56).

Pituitary adenylate cyclase-activating polypeptide (PACAP) and vasoactive intestinal peptide (VIP) complete this convergence. High-affinity PAC1/VIP receptors are present on patient-derived GBM cultures; PACAP/VIP exposure modulates cAMP-EPAC-Rap1 and MAPK cascades, finely balancing proliferation and motility in a context-dependent manner. In hypoxia (pO_2_ ≤ 1%), VIP-VPAC1 signalling curtails EGFR transcription and suppresses Rac1-dependent motility, yet the same pathway under normoxic conditions activates a cAMP-EPAC1-Rap1 cascade that loosens cadherin adhesion and accelerates migration—underscoring a hypoxia-to-normoxia flip from anti- to pro-migratory behaviour (57).

Downstream, these receptors converge on a protease-rich programme that sculpts the peritumoural matrix. NK1-R and CLR activation heighten transcription and surface trafficking of gelatinases (MMP-2/-9) and MT-MMPs, while concurrently up-regulating αvβ3 integrin and focal-adhesion kinase phosphorylation, which together enable traction through dense parenchyma (52, 56, 58). Targeting this redox-sensitive MT1-MMP hub—activated by migraine-associated ROS—offers combined anti-invasive and anti-angiogenic benefit (58).

Neuropeptide signalling also remodels the immune and vascular niches that guide glioma dispersal. SP induces astrocytoma and microglial release of IL-6, IL-8 and GM-CSF, cytokines that polarise tumour-associated macrophages toward an M2-like, invasion-supportive phenotype and stimulate endothelial VEGF secretion. Single-cell RNA-seq atlases nevertheless identify KIT+ TPSAB1+ mast-cells in < 2% of immune cells at the invasive edge, hinting that a sparse but responsive mast-cell niche may still release histamine and VEGF in reaction to SP or CGRP. In parallel, CGRP-family peptides relax peri-tumoural arterioles, augmenting shear stress and facilitating perivascular migration of tumour cells (59, 60). The resulting feedback between nociceptive neuropeptides, immunocytes and vasculature mirrors the sterile inflammation that sensitises meningeal nociceptors during migraine, but in GBM it is repurposed to carve permissive migratory tracks. Recent connectomes in adult GBM and diffuse midline/paediatric high-grade gliomas show CGRP-positive trigeminal afferents forming AMPA-like synapses that drive calcium-dependent invasion (7, 16).

## 4 Bridging evidence between migraine and glioblastoma

The epidemiological intersection between episodic migraine and malignant glioma, while subtle, is increasingly discernible. A nationwide, population-based case-control analysis of more than 22–000 adults showed that patients subsequently diagnosed with brain tumours were 2.45-times more likely to have carried a prior migraine diagnosis; the association remained significant after excluding migraines recorded within three years of tumour detection and was especially pronounced in men (odds ratio 3.04) (59). By contrast, a 39 534-participant prospective cohort of female health professionals that relied on self-reported headache phenotypes failed to detect a higher long-term incidence of brain tumours among migraineurs—a discrepancy the authors ascribed to mis-classification and the small number of high-grade tumours captured over 15 years of follow-up (60). Clinically, headache itself is a sentinel manifestation of glioma; a systematic review encompassing 32 studies reported a weighted mean prevalence of 27% across the disease trajectory, ranking it just behind seizures and cognitive change (61). These data—despite potential confounders such as glucocorticoids, anticonvulsants or anti-VEGF therapy—suggest trigeminovascular activation may precede glioma expansion.

Neuro-imaging observations lend anatomical plausibility to this overlap. High-resolution MRI increasingly delineates glioblastoma growth along cisternal and cavernous segments of the trigeminal nerve as well as Meckel’s cave, underscoring the tumour’s capacity to exploit perineural corridors innervated by migraine-relevant afferents (62). Such tracking brings glioma cells into juxtaposition with meningeal vessels and dural mast cells—the very structures targeted during neurogenic inflammation—creating a micro-environment saturated with vasoactive peptides and proteases. Microdialysis and immuno-electron microscopy indicate that CGRP released from perivascular trigeminal endings at the tumour rim dominates this milieu, whereas intraparenchymal cortical fibres contribute less but may facilitate newly discovered neuron-to-tumour synapses.

At the molecular interface, glioblastoma co-opts virtually every neuropeptide axis canonically implicated in migraine. Transcriptomic and proteomic surveys confirm over-expression of tachykinin and calcitonin-family G-protein-coupled receptors, with full-length neurokinin-1 receptor (NK1-R), PAC1, VPAC1/2 and CLR–RAMP isoforms enriched at the invasive margin (63, 64). Functional studies demonstrate that exogenous substance P accelerates GBM chemotaxis, while radiolabelled SP analogues accumulate selectively within tumour parenchyma (65). Selective NK1-R antagonists (e.g., MEN 11467, aprepitant) produce dose-dependent suppression of U373-MG xenograft growth and trigger apoptotic signalling *in vitro*, directly implicating the SP–NK1-R axis as a driver of invasion rather than a mere epiphenomenon (66). Parallel pathways operate through the CGRP/adrenomedullin family: glioma specimens up-regulate adrenomedullin-2, whose ligation of CLR–RAMP2 boosts ERK1/2 activation, enhances filopodia formation and potentiates temozolomide resistance (49). PACAP and its high-affinity PAC1 receptor are likewise detectable in astrocytic tumours; PACAP modulates cyclic-AMP and MAPK cascades to yield context-dependent trophic or anti-proliferative effects (67, 68).

Pharmacological experience from the migraine field further strengthens this bridge. Aprepitant—licensed for chemotherapy-induced emesis and explored for refractory migraine—induces apoptosis in NK1-R-positive glioma cultures and diminishes Akt phosphorylation *in vivo* (66, 69, 70). Although gepant-class CGRP antagonists have yet to enter neuro-oncology trials, the demonstrable dependence of ADM/CGRP-responsive glioma sub-populations on CLR signalling nominates these agents as credible dual-purpose therapeutics. The emerging discipline of cancer neurobiology recognises sensory neurons as active architects of tumour behaviour, providing a conceptual scaffold that unifies migraine neurogenic inflammation with glioblastoma invasion (71).

Taken together, epidemiological signals, radiological patterns and convergent neuropeptide circuitry converge on a common narrative: the trigeminovascular system and its inflammatory mediators do not merely coexist with glioblastoma but actively facilitate its permeation through neural and perivascular channels.

## 5 Therapeutic perspectives and future directions

A growing body of translational evidence positions the trigeminal neuropeptide axis as more than an epiphenomenon of tumour-induced pain; instead, it represents a tractable vulnerability that can be co-targeted to blunt glioblastoma (GBM) infiltration while simultaneously alleviating migraine-like symptomatology. The convergence of CGRP, substance P, PACAP and adrenomedullin signalling on matrix-remodelling, angiogenesis and immune polarisation suggests that pharmacological or radiopharmaceutical interruption of these cues could deliver a double therapeutic dividend.

Repurposing clinically approved antagonists is the most immediate path to bedside impact. Yet BBB pharmacokinetics diverge sharply: anti-CGRP monoclonal antibodies (~150 kDa) cross an intact barrier at < 0.1% ID, restricting action to regions of contrast enhancement, whereas the lipophilic NK1-antagonist aprepitant (MW ≈ 534 Da, logP ≈ 3.5) reaches CSF-to-plasma ratios of ~0.05—adequate for receptor occupancy but still sub-therapeutic in deeply infiltrated zones. Moreover, chronic CGRP blockade erodes vasodilatory reserve, slows dermal repair, and may blunt dendritic priming—risks that must be balanced during multimodal therapy. Aprepitant, an oral neurokinin-1 receptor (NK1-R) blocker widely used for chemotherapy-induced nausea, induces apoptosis, suppresses lamellipodial dynamics and synergises with 5-aminolevulinic acid in patient-derived GBM cultures (72, 73). Compassionate-use series and the multi-drug CUSP9 protocol already exploit this agent in recurrent disease, providing a safety dossier that greatly exceeds that of *de-novo* anticancer compounds. Parallel interest surrounds gepant CGRP antagonists now used for migraine; however, migraine doses yield sub-micromolar intratumour exposure, so carrier-mediated delivery or escalation will be necessary to exploit CLR/RAMP2–3 dependency (74, 75).

Where systemic small molecules may falter against the blood-brain barrier, peptide-based radiotheranostics offer focal, high-linear-energy transfer delivery. Targeted α-therapy with NK1-directed peptides has yielded encouraging but still preliminary data: the largest 225Ac-DOTA-Substance P dose-escalation study (n = 21, median 3 cycles) reported grade-3 thrombocytopenia in 1/21 patients and a median overall survival (OS) of 9.0 months from first dose, with RANO responses limited to one partial response and seven cases of stable disease. An earlier 213Bi-DOTA-Substance P trial (n = 20, cumulative activity ≤ 11.2 GBq) recorded only transient grade 1/2 toxicities and a 7.5-month median OS from therapy start, again with low objective-response rates (76, 77). Updated European Association of Nuclear Medicine guidelines now list NK1-R–targeted alpha therapy among the most mature investigational options for infiltrative glioma (78, 79), while complementary β-emitting constructs are entering first-in-human evaluation. Such vector-agnostic platforms could, in principle, be adapted to CGRP or PACAP receptors as radioligand scaffolds once high-affinity ligands become available.

Adrenomedullin, a close CGRP family member, exemplifies the broader potential of neuropeptide blockade. Neutralising antibodies or small-molecule antagonists down-regulate JNK–cyclin-D1 signalling, curb spheroid expansion and enhance temozolomide sensitivity in xenografts (80, 81). Given its additional roles in vascular permeability, dual adrenomedullin/VEGF inhibition could synergistically normalise aberrant tumour vessels, thereby improving immune-cell ingress and drug delivery.

Future development will hinge on precise patient stratification. Receptor-specific PET tracers (e.g., ^68^Ga-labelled NK1-R or uPAR ligands) already delineate invasive margins with sub-centimetre resolution and could guide convection-enhanced delivery catheters or focused-ultrasound BBB modulation. Integration of these imaging biomarkers with plasma neuropeptide signatures promises real-time pharmacodynamic read-outs and early identification of escape pathways. Coupling neuropeptide blockade with immunotherapy is mechanistically attractive because CGRP- and SP-driven M2 polarisation up-regulates PD-L1; neutralising these cues may re-programme macrophages and rest.
